# Supplementary material for: Systems biology analysis uncovers a ROS-associated gene signature and immunomodulatory role of CLEC4E in ischemic stroke
Source: PLoS One. 2026 Mar 10;21(3):e0344443. doi: 10.1371/journal.pone.0344443 (PMC12974805; doi:10.1371/journal.pone.0344443)
Supplement: S1 Table — (DOCX) [file pone.0344443.s001.docx]

**Table S1.** siRNA sequences for CLEC4E.

| Gene | sense 5’-3’ | antisense 5’-3’ |
| --- | --- | --- |
| si-NC | UUCUCCGAACGUGUCACGUUU | ACGUGACAGGUUCGGAGAAUU |
| si-CLEC4E-1 | UUGAAAGAUGCGAAAUGUCAC | GACAUUUCGCAUCUUUCAAAC |
| si-CLEC4E-2 | AAUAUUCCCAGUUCAAUGGAC | CCAUUGAACUGGGAAUAUUUU |
| si-CLEC4E-3 | AGAUGAUUUAGAUGAAUUCAU | GAAUUCAUCUAAAUCAUCUGA |
