# Supplementary material for: Systems biology analysis uncovers a ROS-associated gene signature and immunomodulatory role of CLEC4E in ischemic stroke
Source: PLoS One. 2026 Mar 10;21(3):e0344443. doi: 10.1371/journal.pone.0344443 (PMC12974805; doi:10.1371/journal.pone.0344443)
Supplement: S2 Table — (DOCX) [file pone.0344443.s002.docx]

**Table S2.** Primers for qRT-PCR.

| Gene | Forward | Reverse |
| --- | --- | --- |
| CLEC4E-human | CTGAAACACAATGCACAGAGAGA | AAAGATGCGAAATGTCACAACAC |
| TNF-α-human | GCCTGTACCTCATCTACTC | CCTTGGTCTGGTAGGAGA |
| IL-1β-human | CTGTCGTGCGTGTTGAAAGA | TTCTGCTTGAGAGGTGCTGA |
| IL-6-human | GACAGCCACTCACCTCTTCA | AGTGCCTCTTTGCTGCTTTC |
| GAPDH-human | GGAGCGAGATCCCTCCAAAAT | GGCTGTTGTCATACTTCTCATGG |
| CLEC4E-mouse | AGTGCTCTCCTGGACGATAG | CCTGATGCCTCACTGTAGCAG |
| SLC8A1-mouse | CTTCCCTGTTTGTGCTCCTGT | AGAAGCCCTTTATGTGGCAGTA |
| HIST1H4H-mouse | CCTGTGCCTTCCACTCTGTT | TGTTGTCACGCAGCACTTTG |
| BMX-mouse | TACCTGGCTGAAAACTACTGCT | CCACATCATATTGCCCCTTCCA |
| MCEMP1-mouse | CGGTGTGGAATATCCGGGAG | TCATGACTGTGCCAAGGGTC |
| KREMEN1-mouse | ACGGTGCAGATTACAGGGGA | TTGGGGTACTTCAGCGTGTTG |
| ZFP36L2-mouse | AGCGGCTCCCAGATCAACT | CGAAAGCGAAGGCGTTGTTA |
| GAPDH-mouse | AGGTCGGTGTGAACGGATTTG | TGTAGACCATGTAGTTGAGGTCA |
