# Supplementary material for: Systems biology analysis uncovers a ROS-associated gene signature and immunomodulatory role of CLEC4E in ischemic stroke
Source: PLoS One. 2026 Mar 10;21(3):e0344443. doi: 10.1371/journal.pone.0344443 (PMC12974805; doi:10.1371/journal.pone.0344443)
Supplement: S3 File — (PDF) [file pone.0344443.s003.pdf]

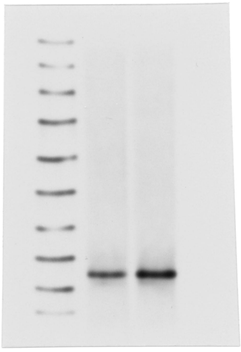

Figure legend. The western blot results of CLEC4E protein in the sham and IS group.

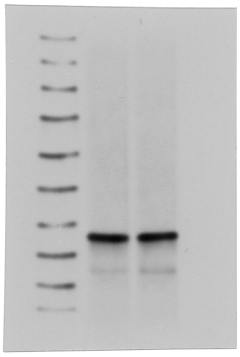

Figure legend. The western blot results of GAPDH protein in the sham and IS group.
