# Supplementary material for: Systems biology analysis uncovers a ROS-associated gene signature and immunomodulatory role of CLEC4E in ischemic stroke
Source: PLoS One. 2026 Mar 10;21(3):e0344443. doi: 10.1371/journal.pone.0344443 (PMC12974805; doi:10.1371/journal.pone.0344443)
Supplement: Attachment — Submitted filename: Reviewer s reply.pdf [file pone.0344443.s004.pdf]

In this paper, we reveal the role of ROS-related gene features in ischemic stroke through systems biology methods, and propose CLEC4E as a potential immune regulator, which is innovative. However, there are some problems to be solved.

1. Supplementary method details
Specify the sample size of the cohort, quality control (PCA analysis), data preprocessing steps (such as batch effect correction, whether the data is preprocessed), and WGCNA module definition threshold (such as dynamic shear tree height).

2. The WGCNA in Figure 2 is an analysis using the GSE58294 or GSE16561 cohort. Please describe it clearly.

Which cohort was used for the LASSO regression analysis in Figure 4?

Please describe which cohort was used for the immune infiltration analysis in Figure 7?

3. The study fails to thoroughly explore how CLEC4E connects ROS with immune infiltration (e.g., receptor-ligand interactions and downstream signaling pathways). It is necessary to supplement the comparison with existing biomarkers (e.g., NSE and S100B).

4. The potential applications of gene signatures in early diagnosis, prognostic stratification, or therapeutic target development were not discussed. A comparison with existing biomarkers (e.g., NSE and S100B) should be supplemented.

5. Human Data Ethics Statement : The document fails to specify whether the GEO data has undergone ethical review. A supplementary statement regarding the ethical compliance of data sources should be included.

6. Completeness of supplementary materials : While uncropped Western blot images are labeled as "not for publication", critical experimental data should be made publicly available to ensure reproducibility. It is recommended that these key data be fully presented in the supplementary materials.

7. Please provide the code for all analysis.
