# Supplementary material for: Systems biology analysis uncovers a ROS-associated gene signature and immunomodulatory role of CLEC4E in ischemic stroke
Source: PLoS One. 2026 Mar 10;21(3):e0344443. doi: 10.1371/journal.pone.0344443 (PMC12974805; doi:10.1371/journal.pone.0344443)
Supplement: Attachment — Submitted filename: Response to Reviewers.docx [file pone.0344443.s006.docx]

**Manuscript Number:** PONE-D-25-51535

**Title:** Systems Biology Analysis Uncovers a ROS-Associated Gene Signature and Immunomodulatory Role of CLEC4E in Ischemic Stroke

From:

**Professor Xiaodi Ding**

Center for Rehabilitation Medicine, Rehabilitation & Sports Medicine Research Institute of Zhejiang Province, Department of Rehabilitation Medicine, Zhejiang Provincial People’s Hospital(Affiliated People’s Hospital), Hangzhou Medical College, Hangzhou, 310014, Zhejiang, China.

January 9th, 2026

**Dear Editor and reviewers:**

Thank you very much for your detailed constructive and active comments on our manuscript.

According to each comment from reviewers, we have revised the manuscript carefully and made corresponding changes point by point. All the changes including the text and tables have been added and labeled in yellow in the revised manuscript according to this response. The list of changes has been itemized and explained in the response to reviewers.

Thanks again for your constructive suggestions. Thanks for your kind consideration of our article again. If you have any questions about the manuscript, please feel free to contact me. Merry Christmas!

Sincerely yours,

**Professor Xiaodi Ding**

E-mail: Qiwenlong11588@126.com

### Responses to Journal requirements：

**Comment 1.** Please ensure that your manuscript meets PLOS ONE's style requirements, including those for file naming. The PLOS ONE style templates can be found at https://journals.plos.org/plosone/s/file?id=wjVg/PLOSOne_formatting_sample_main_body.pdf and https://journals.plos.org/plosone/s/file?id=ba62/PLOSOne_formatting_sample_title_authors_affiliations.pdf

**Response**: Thank you for this valuable suggestion. We have made revisions in accordance with the style requirements of the PLOS ONE.

2. To comply with PLOS One submissions requirements, in your Methods section, please provide additional information regarding the experiments involving animals and ensure you have included details on (1) methods of sacrifice, (2) methods of anesthesia and/or analgesia, and (3) efforts to alleviate suffering.

**Response**: Thank you for this valuable suggestion. We have made detailed supplements to the animal experiment methods.

3. Thank you for stating the following financial disclosure:

“This work was supported by the Zhejiang Provincial Administration of Traditional Chinese Medicine plans (Grant No. C-2025-W219).”

Please state what role the funders took in the study. If the funders had no role, please state: "The funders had no role in study design, data collection and analysis, decision to publish, or preparation of the manuscript."

If this statement is not correct you must amend it as needed.

Please include this amended Role of Funder statement in your cover letter; we will change the online submission form on your behalf.

**Response**: Thank you for this valuable suggestion. “This work was supported by the Zhejiang Provincial Administration of Traditional Chinese Medicine plans (Grant No. C-2025-W219). The funders had no role in study design, data collection and analysis, decision to publish, or preparation of the manuscript.” We have included this amended Role of Funder statement in our cover letter.

4. Thank you for stating the following in the Funding Section of your manuscript:

“This work was supported by the Zhejiang Provincial Administration of Traditional Chinese Medicine plans (Grant No. C-2025-W219).”

We note that you have provided funding information that is currently declared in your Funding Statement. However, funding information should not appear in the Acknowledgments section or other areas of your manuscript. We will only publish funding information present in the Funding Statement section of the online submission form.

Please remove any funding-related text from the manuscript and let us know how you would like to update your Funding Statement. Currently, your Funding Statement reads as follows:

“This work was supported by the Zhejiang Provincial Administration of Traditional Chinese Medicine plans (Grant No. C-2025-W219).”

Please include your amended statements within your cover letter; we will change the online submission form on your behalf.

**Response**: Thank you for this valuable suggestion. We have removed any funding-related text from the manuscript and included the amended Role of Funder statement in our cover letter.

“This work was supported by the Zhejiang Provincial Administration of Traditional Chinese Medicine plans (Grant No. C-2025-W219). The funders had no role in study design, data collection and analysis, decision to publish, or preparation of the manuscript.”

5. PLOS requires an ORCID iD for the corresponding author in Editorial Manager on papers submitted after December 6th, 2016. Please ensure that you have an ORCID iD and that it is validated in Editorial Manager. To do this, go to ‘Update my Information’ (in the upper left-hand corner of the main menu), and click on the Fetch/Validate link next to the ORCID field. This will take you to the ORCID site and allow you to create a new iD or authenticate a pre-existing iD in Editorial Manager.

**Response**: Thank you for this valuable suggestion. I have linked my ORCID in the system.

6. Your ethics statement should only appear in the Methods section of your manuscript. If your ethics statement is written in any section besides the Methods, please move it to the Methods section and delete it from any other section. Please ensure that your ethics statement is included in your manuscript, as the ethics statement entered into the online submission form will not be published alongside your manuscript.

**Response**: Thank you for this valuable suggestion. We have moved our ethics statement to the Methods section and delete it from any other section.

7. PLOS ONE now requires that authors provide the original uncropped and unadjusted images underlying all blot or gel results reported in a submission’s figures or Supporting Information files. This policy and the journal’s other requirements for blot/gel reporting and figure preparation are described in detail at https://journals.plos.org/plosone/s/figures#loc-blot-and-gel-reporting-requirements and https://journals.plos.org/plosone/s/figures#loc-preparing-figures-from-image-files. When you submit your revised manuscript, please ensure that your figures adhere fully to these guidelines and provide the original underlying images for all blot or gel data reported in your submission. See the following link for instructions on providing the original image data: https://journals.plos.org/plosone/s/figures#loc-original-images-for-blots-and-gels.

In your cover letter, please note whether your blot/gel image data are in Supporting Information or posted at a public data repository, provide the repository URL if relevant, and provide specific details as to which raw blot/gel images, if any, are not available. Email us at plosone@plos.org if you have any questions.

**Response**: Thank you for this valuable suggestion. We have uploaded the Uncropped western blots to the system as supplementary material as required.

8. Please include captions for your Supporting Information files at the end of your manuscript, and update any in-text citations to match accordingly. Please see our Supporting Information guidelines for more information: http://journals.plos.org/plosone/s/supporting-information.

If the reviewer comments include a recommendation to cite specific previously published works, please review and evaluate these publications to determine whether they are relevant and should be cited. There is no requirement to cite these works unless the editor has indicated otherwise.

**Response**: Thank you for this valuable suggestion. We have made the modifications as required.

### Responses to Reviewer #1

In this paper, we reveal the role of ROS-related gene features in ischemic stroke through systems biology methods, and propose CLEC4E as a potential immune regulator, which is innovative. However, there are some problems to be solved.

**Comment 1.** Supplementary method details

Specify the sample size of the cohort, quality control (PCA analysis), data preprocessing steps (such as batch effect correction, whether the data is preprocessed), and WGCNA module definition threshold (such as dynamic shear tree height).

**Response**: Thank you for your valuable feedback. In response to your request, we have added the following key methodological details: (1). Sample sizes: The GSE58294 cohort includes 69 ischemic stroke (IS) samples and 23 normal controls; the GSE16561 cohort contains 39 IS samples and 24 normal controls. (2). Quality control and data preprocessing: Principal component analysis (PCA) was conducted for sample quality assessment. All samples exhibited acceptable clustering within their respective diagnostic groups, and no samples were excluded. Raw expression data were background-corrected and normalized using the limma package in R, with probes mapped to gene symbols based on platform annotation files. Since the two datasets were analyzed independently (e.g., within-cohort comparisons such as IS vs. control), systematic cross-dataset batch correction was not applied. 3.WGCNA parameter settings: A soft-thresholding power (β = 12) was selected to meet the scale-free topology criterion, which ensures the constructed network has biological rationality. Modules were identified through hierarchical clustering with the following parameters: a minimum module size of 30 genes, and a merge cut height of 0.25.

These details have been incorporated into Sections 2.1 and 2.4 of the manuscript, enhancing the transparency and reproducibility of our methodological approach.

**Comment 2.** The WGCNA in Figure 2 is an analysis using the GSE58294 or GSE16561 cohort. Please describe it clearly.

Which cohort was used for the LASSO regression analysis in Figure 4?

Please describe which cohort was used for the immune infiltration analysis in Figure 7?

**Response**: Thank you for raising these important points about cohort usage. We have provided the following clarifications: 1. The weighted gene co-expression network analysis (WGCNA) in Figure 2 was performed based on the GSE58294 cohort data. In this study, GSE58294 was used as the discovery set, and module preservation analysis was conducted via an internal sample stratification strategy (75% training set, 25% test set) to verify the robustness of co-expression modules. 2. The LASSO regression model (Figure 4) was trained on GSE58294 and independently validated on GSE16561. 3. Immune infiltration analysis (Figure 7) was performed on GSE58294 to maintain analytical consistency with the preceding steps.

These details have been integrated into the relevant method sections (2.4 and 2.7) to enhance clarity.

**Comment 3.** The study fails to thoroughly explore how CLEC4E connects ROS with immune infiltration (e.g., receptor-ligand interactions and downstream signaling pathways). It is necessary to supplement the comparison with existing biomarkers (e.g., NSE and S100B).

**Response**: Thank you for this valuable feedback. In response, we have expanded the Discussion section to address these points by: (1) detailing the potential molecular mechanisms linking CLEC4E to ROS‑immune crosstalk, including receptor‑ligand interactions and downstream signaling; and (2) providing a systematic comparison between our CLEC4E‑based signature and established biomarkers such as NSE and S100B.

**Comment 4.** The potential applications of gene signatures in early diagnosis, prognostic stratification, or therapeutic target development were not discussed. A comparison with existing biomarkers (e.g., NSE and S100B) should be supplemented.

**Response**: Thank you for this valuable suggestion. In response, we have expanded the Discussion to address clinical applications and comparisons with existing biomarkers:

Traditional ischemic stroke biomarkers (e.g., NSE, S100B), which primarily reflect structural damage to neurons and glial cells, present several limitations: (1) insufficient sensitivity in the hyperacute phase, making early warning challenging; (2) inability to differentiate etiological subtypes of stroke; (3) limited to assessing injury severity without reflecting dynamic pathological processes such as immune inflammation. In contrast, the CLEC4E-based signature developed in this study dynamically captures the interactive state between oxidative stress and immune-inflammatory networks at the transcriptional level. This not only offers new potential for hyperacute diagnosis but also enables the identification of “high-oxidation, high-inflammation” patient subtypes through its scoring system, guiding personalized targeted therapies. More importantly, as a druggable cell-surface receptor, CLEC4E provides a direct target for developing monoclonal antibodies or small-molecule inhibitors, establishing a translational closed loop from diagnostic marker to therapeutic target.

**Comment 5.** Human Data Ethics Statement: The document fails to specify whether the GEO data has undergone ethical review. A supplementary statement regarding the ethical compliance of data sources should be included.

**Response**: We thank the reviewer for raising this important point regarding ethical compliance. All data used in this study were obtained from the publicly available Gene Expression Omnibus (GEO) database (accession numbers: GSE58294 and GSE16561). As stated on the GEO platform and in the original publications citing these datasets, the data were collected in accordance with the ethical standards of the respective originating institutions, including obtaining informed consent from all participants and approval from the relevant institutional review boards (IRBs). A statement clarifying this has been added to the Methods section (Section 2.1).

**Comment 6.** Completeness of supplementary materials: While uncropped Western blot images are labeled as “not for publication”, critical experimental data should be made publicly available to ensure reproducibility. It is recommended that these key data be fully presented in the supplementary materials.

**Response**: We sincerely thank the reviewer for this important suggestion regarding data transparency. We have fully presented these key data in the supplementary materials. We believe these measures will significantly enhance the transparency and verifiability of our study.

**Comment 7.** Please provide the code for all analysis.

**Response**: Thank you for the suggestion. We have uploaded all the raw data to Science Data Bank (Data private access link: https://www.scidb.cn/en/s/zmeyMb), and the code for all analysis as a supplementary file upload system, to ensure full transparency and reproducibility.

### Responses to Reviewer #2

This manuscript integrates two independent transcriptomic cohorts to map reactive oxygen species (ROS)–related programs in ischemic stroke and to derive a compact gene signature with diagnostic/predictive utility, complemented by immune-deconvolution analyses and in vivo validation.

**Comment 1.** Please provide a more explicit description of each GEO cohort (sample sizes, tissue/source, timing relative to stroke onset, key clinical covariates, and inclusion/exclusion criteria), as these factors can materially influence transcriptomic patterns and immune estimates. Also clarify how potential batch effects across platforms/cohorts were handled (or why not needed), how missing clinical information was operationalized, and whether any confounder adjustment was performed beyond quality control.

**Response**: Thank you for raising these important points regarding the detailed description of the GEO cohorts and our analytical approach. We appreciate the opportunity to clarify these methodological aspects. Below, we provide a summary of the relevant information based on the descriptions already included in the Methods section 2.1.

(1). GSE58294 contains peripheral blood samples from 69 ischemic stroke (IS) patients collected at three time points (within 3 hours, at 5 hours, and at 24 hours post-onset) and 23 healthy controls. GSE16561 consists of whole-blood samples from 39 IS patients and 24 healthy controls.

(2). Raw expression data from both cohorts were background-corrected and normalized using the limma package in R, with probes mapped to official gene symbols based on the respective platform annotation files. Principal component analysis (PCA) was performed for sample quality assessment, and all samples demonstrated acceptable clustering within their respective diagnostic groups. No samples were excluded. Regarding potential batch effects between the two cohorts, systematic cross-dataset batch correction was not applied because the two datasets were analyzed independently in this study.

We hope this clarification addresses your concerns. Should further details or adjustments be needed, we would be glad to provide them. Thank you again for your valuable feedback.

**Comment 2.** The analysis is anchored to five curated ROS-related gene sets; however, readers will benefit from a clear rationale for choosing these specific sets and how overlapping genes across sets were handled. Consider adding sensitivity analyses using alternative ROS resources (or broader oxidative-stress annotations) and report whether the key module and the final gene signature remain stable under reasonable variations in ROS gene set definitions.

**Response:**

**1) Response to Reviewer’s Question about the Selection of ROS-Related Gene Sets:**

Thank you for your valuable comments. As the reviewer pointed out, the selection of ROS-related gene sets is a critical foundation of this study, and we carefully considered this issue during the initial design of our research. Our overall approach is outlined below:

1. **Selection of Core ROS-Related Gene Sets**: We prioritized the inclusion of genes that are directly involved in ROS-related pathways, rather than relying on general associations based on common literature or databases (such as GeneCards). We believe that focusing on gene sets directly related to ROS is essential for studying the role of ROS in ischemic stroke.
2. **Selection of ROS Pathways from MSigDB**: Among the various available resources, MSigDB is widely used in ROS research and includes multiple ROS-related pathways. To ensure that the selected gene sets are representative and authoritative, we chose five key ROS pathways from MSigDB.
3. **Avoidance of Indirect Pathways**: To maintain the focus of our research, we intentionally avoided pathways that indirectly regulate ROS through other processes. Our study is centered on ROS itself, so we excluded pathways that modulate ROS indirectly and only selected the most directly relevant ROS pathways.
4. **Handling Gene Set Overlap**: As the reviewer mentioned, there is some overlap in genes across these ROS-related pathways. However, it is important to note that the genes within each pathway do not overlap with one another. Therefore, there was no need to perform gene deduplication in our analysis. The selected gene sets ensure that the most core and compelling genes are included, and the subsequent WGCNA analysis further selected the most relevant modules, ensuring the accuracy of the analysis.

In summary, by selecting the most core and directly ROS-related gene sets from MSigDB and rigorously filtering them, we believe the chosen gene sets provide a solid and scientifically valid foundation for studying the role of ROS in ischemic stroke. We have provided additional clarifications in the Methods.

**2) Response to Reviewer’s Question about Module Stability and Sensitivity Analysis:**

Thank you for your insightful comment. As part of our analysis, we have indeed performed a series of steps to ensure the stability of the identified modules. Specifically, we conducted module preservation analysis to assess the robustness of the gene modules across different datasets.

In the analysis, we used the modulePreservation function from the WGCNA package, which allows us to evaluate the stability of modules by comparing their preservation across independent training and test datasets. This process ensures that the identified modules are robust and reproducible, even under different conditions. We also performed statistical evaluations of module preservation, including median rank and Z-summary, to confirm that the modules are consistently preserved across datasets (***Figure 2D, n=200***).

Additionally, we used module eigengene analysis to assess the association between modules and traits, and visualized the module-trait relationships to further validate the relevance of the modules. These analyses confirm that the modules, especially the core ROS-related modules, are stable and reproducible.

We have attached the original data files and the R code used in our analysis for your reference. This includes all necessary steps for module preservation analysis, module-trait relationships, and the generation of relevant plots, ensuring transparency and reproducibility of our findings.

Thank you again for your thoughtful review. We are confident that the stability of the modules has been carefully validated, and we hope this additional information addresses your concern.

**Comment 3.** Because WGCNA results can vary with parameter settings, please report key choices (soft-threshold selection criteria, minimum module size, merge thresholds) and provide more explicit module preservation outputs. It would also help to clarify whether the network was constructed on all genes vs. ROS-filtered genes (and the trade-offs), and to demonstrate that the “brown module” association is not driven by cohort-specific artifacts.

**Response:** Thank you for raising these important points regarding the methodological details and robustness of our WGCNA analysis. We appreciate your suggestions and have addressed them as follows:

(1). WGCNA parameter settings: A soft-thresholding power (β = 12) was selected to meet the scale-free topology criterion, which ensures the constructed network has biological rationality. Modules were identified through hierarchical clustering with the following parameters: a minimum module size of 30 genes, and a merge cut height of 0.25. The WGCNA network was built using the ROS-related gene subset obtained by merging the five predefined ROS gene sets.

(2). To verify the robustness of the co-expression modules identified in the GSE58294 cohort, we adopted an internal sample stratification strategy and performed module preservation analysis using the modulePreservation function, with 20 permutation tests (nPermutations = 20). The training set (75% of samples) served as the reference network to validate module preservation in the test set (25% of samples). As shown in Figure 2D, the key brown module achieved a high Z-summary value of 60 in the test set, significantly exceeding the empirical threshold for strong preservation (Z-summary > 10), with a top-ranked median rank, indicating good preservation across sample subgroups. Module-trait relationship analysis revealed a significant and stable correlation between the brown module and target traits in the full dataset. Additionally, the brown module contained a sufficient number of genes, ensuring robust statistical power to avoid false positives caused by cohort-specific artifacts. Collectively, these results confirm that the co-expression pattern of the brown module is not a dataset-specific artifact but a biologically stable feature conserved across sample subgroups.

We have incorporated these clarifications and additional analyses into Sections 2.4 and 2.5 of the Methods.

**Comment 4.** The manuscript would be strengthened by a clearer statement of the prediction target (diagnosis vs. risk vs. prognosis) and a consistent modeling description. Ensure that feature selection, model fitting, and hyperparameter tuning are fully nested within cross-validation to avoid optimistic bias. In addition to AUC, please report clinically relevant metrics (e.g., sensitivity/specificity at prespecified thresholds, PPV/NPV with plausible prevalence, and calibration diagnostics) and justify how decision-curve analysis was parameterized for this setting.

**Response:** Thank you for your constructive comments. We have carefully revised the manuscript to address the issues raised regarding the prediction target, model description, and clinical metrics. Below are our detailed responses:

1. **Prediction Target Clarification**: Based on your suggestion, we have updated the manuscript to clearly define our model as a diagnostic model. The primary goal of our model is to differentiate between ischemic stroke (IS) and control groups based on ROS-related gene expression, making it a diagnostic tool. This change in terminology has been reflected throughout the manuscript to ensure consistency.
2. **Modeling Process and Cross-Validation**: We have ensured that all steps of feature selection, model fitting, and hyperparameter tuning are fully nested within cross-validation to avoid optimistic bias. Specifically, we performed repeated cross-validation to evaluate the robustness of our model, ensuring that the process of model training and validation was not biased by data leakage or overfitting. The out-of-fold AUC distributions from the repeated cross-validation demonstrated consistent performance across datasets, with mean AUC values of 0.81 in GSE58294 and 0.86 in GSE16561, confirming the model's robustness (Figure 5C-D).
3. **Clinical Metrics**: In addition to the commonly reported AUC, we have conducted extensive validation of the predictive performance. Although the publicly available datasets do not include additional clinical metrics (such as sensitivity, specificity, PPV/NPV, and prevalence), we have made efforts to ensure the clinical relevance of the results in the following ways:
   - **Calibration Curves**: We reported calibration curves to evaluate the agreement between predicted and observed outcomes, which showed excellent concordance in GSE58294 (C-index = 0.995) and acceptable agreement in GSE16561 (C-index = 0.717) (Figure 5E-F).
   - **Decision Curve Analysis (DCA)**: We performed decision-curve analysis to assess the net clinical benefit of the risk score model compared to “treat all” or “treat none” strategies. This analysis demonstrated that the risk score model provides greater clinical benefit across a wide range of threshold probabilities (Figure 5G-H). The decision curve was parameterized based on clinically relevant thresholds and modeled against these strategies, which aligns with the clinical utility of the model.

We acknowledge that the lack of clinical data in the publicly available datasets limits the direct calculation of some clinical metrics (e.g., sensitivity, specificity, and NPV/PPV with plausible prevalence). However, we have included the most relevant and widely accepted metrics (AUC, calibration, and decision-curve analysis) to provide a comprehensive evaluation of the model’s predictive performance.

Thank you once again for your valuable feedback. We hope that the revisions and clarifications we have made adequately address your concerns and strengthen the manuscript.

**Comment 5.** Deconvolution methods can diverge depending on signatures and tissue context. Please quantify concordance across algorithms, discuss assumptions (especially if derived from blood expression profiles), and consider additional checks (e.g., whether observed correlations persist after controlling for global inflammation markers). The negative association between CLEC4E and B/T cell infiltration is intriguing, but causal language should be avoided without mechanistic experiments.

**Response:** Thank you for your insightful comments regarding the immune infiltration analysis. We appreciate your attention to the complexities of deconvolution methods, and we have taken the following actions to address the concerns you raised:

1. **Concordance Across Algorithms**: As you rightly pointed out, deconvolution methods can produce divergent results depending on the signatures and tissue context. In our analysis, we included multiple algorithms (MCPcounter, quanTIseq, xCell, and EPIC) to ensure a robust and consistent assessment of immune cell infiltration across various methods. By employing different algorithms, we aimed to capture a comprehensive picture of immune infiltration and identify consistent patterns across these approaches. We have quantified the concordance across algorithms and provided a discussion of the agreement between the results obtained from each method.
2. **Discussion of Assumptions**: We acknowledge that deconvolution algorithms, especially those derived from blood expression profiles, may not fully capture tissue-specific nuances. In our study, we used these methods as a proxy for immune infiltration, and we have explicitly discussed this limitation in the revised manuscript. We recognize that the assumption of using blood-based signatures may not fully reflect the immune landscape in ischemic stroke tissue, and we have highlighted this as a potential area of future research. However, these algorithms are commonly used and provide valuable insights into immune cell composition, even if they may have limitations when applied to tissue-specific contexts.
3. **Causal Language Regarding CLEC4E**: Regarding the negative association between CLEC4E expression and B/T cell infiltration, we agree with your point that causal language should be avoided in the absence of mechanistic experiments. While our findings suggest an intriguing negative correlation, we have revised the manuscript to ensure that we do not overstate these results. We now emphasize the associative nature of the relationship between CLEC4E expression and immune infiltration, acknowledging that further experimental validation is needed to explore the underlying mechanisms.

We hope these clarifications and additional analyses address your concerns and enhance the robustness of our findings. We are grateful for your thoughtful suggestions, and we believe they have strengthened the manuscript.

**Comment 6.** It would be useful to briefly situate your pathway/functional enrichment pipeline within the landscape of contemporary large-cohort, reproducible enrichment workflows by adding a few representative citations (e.g., https://doi.org/10.1038/s41467-025-61785-z;https://doi.org/10.1186/s12916-023-02920-9; https://doi.org/10.1503/jpn.220202). This small addition would help readers benchmark your enrichment choices (gene-set resources, multiple-testing control, and interpretation strategy) against established population-scale practice.

**Response:** Thank you very much for this constructive feedback. We fully agree that situating our analytical workflow within established large-scale practices enhances methodological transparency and facilitates better benchmarking. As requested, we have added citations to contemporary large-cohort studies in Methods Sections 2.2 and 2.6 to provide context for our pathway and functional enrichment analysis pipeline. We believe these targeted additions successfully link our methods with contemporary population-scale practices, offering readers a clear framework to assess the robustness of our enrichment analysis. We sincerely appreciate your guidance in helping us improve the academic rigor of our manuscript.

**Comment 7.** In the Statistical analyses section, consider referencing several large-sample database studies that use closely related modeling and validation strategies (e.g., https://doi.org/10.1016/j.dcn.2025.101566;https://doi.org/10.1016/j.psychres.2025.116503; <https://doi.org/10.1192/bjp.2023.107).>

**Response:** We sincerely thank you for this valuable suggestion. In response, we have supplemented the relevant citations in the Statistical Analysis section (Section 2.23). These additions help to demonstrate that our employed strategies—Elastic Net regression, cross-validation, and independent cohort validation—align with established methodological standards in contemporary large-scale studies, thereby further supporting the reliability and advanced nature of our methodological choices.

**Comment 8.** Please provide additional detail on randomization/blinding, sample sizes and power considerations, timepoints, and whether endpoints were prespecified. To better connect the computational findings to mechanism, consider functional perturbation (e.g., knockdown/overexpression or pharmacologic modulation) targeting CLEC4E (or its upstream/downstream signaling) and report whether this alters ROS burden, immune infiltration markers, and neuronal injury outcomes in the model.

We sincerely appreciate the reviewer’s valuable comments. 1. We have now clarified the experimental details in Section 2.19 of the Methods. Randomization was applied during model establishment and analyses. Animals were randomly assigned to groups, and all behavioral, histological, and image analyses were performed under blinded conditions. The sample size was set at 10 mice per group, based on previous similar studies and laboratory practice, which we considered appropriate for this exploratory investigation. The primary endpoint was prespecified as molecular and histological assessment at 24 hours post-modeling, a timepoint selected to capture acute inflammatory, oxidative, and tissue injury responses. All molecular and histological outcome measures were defined before data collection. 2. To establish a robust link between computational findings and mechanistic validation, we have supplemented functional perturbation experiments targeting CLEC4E (siRNA-mediated knockdown) in the SH-SY5Y cell OGD/R model, as presented in Figure 8. These experiments verified that CLEC4E knockdown alleviates neuronal injury, reduces ROS accumulation, and suppresses macrophage M1 polarization and pro-inflammatory cytokine expression. The detailed experimental methods have been added to Sections 2.12-2.18, and the corresponding results are included in Section 3.8 of the revised manuscript. We sincerely appreciate your valuable comment, which has significantly improved the quality of our study.
